# Weighted Uncertainty Relations

**DOI:** 10.1038/srep23201

**Published:** 2016-03-17

**Authors:** Yunlong Xiao, Naihuan Jing, Xianqing Li-Jost, Shao-Ming Fei

**Affiliations:** 1 School of Mathematics, South China University of Technology, Guangzhou 510640, China; 2 Max Planck Institute for Mathematics in the Sciences, Leipzig 04103, Germany; 3 Department of Mathematics, North Carolina State University, Raleigh, NC 27695, USA; 4 School of Mathematical Sciences, Capital Normal University, Beijing 100048, China

## Abstract

Recently, Maccone and Pati have given two stronger uncertainty relations based on the sum of variances and one of them is nontrivial when the quantum state is not an eigenstate of the sum of the observables. We derive a family of weighted uncertainty relations to provide an optimal lower bound for all situations and remove the restriction on the quantum state. Generalization to multi-observable cases is also given and an optimal lower bound for the weighted sum of the variances is obtained in general quantum situation.

In Kennard’s formulation^1^ of Heisenberg’s uncertainty principle^2^, for any single quantum particle, the product of the uncertainties of the position and momentum measurements is at least half of the Planck constant (see also the work of Weyl^3^)


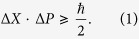


Later Robertson^4^ derived the uncertainty principle for any pair of observables *A* and *B* with bounded spectrums:


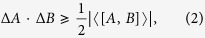


where Δ*A*^2^ = 〈*A*^2^〉 − 〈*A*〉^2^ is the variance of operator *A* over the state |*ψ*〉. Eq. (2) can be derived from a slightly strengthened inequality, the Schrödinger uncertainty relation^5^


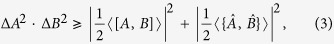


where 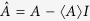 and *I* is the identity operator.

All these inequalities^6^,^7^ can be trivial even if *A* and *B* are incompatible on the state of the system |*ψ*〉, for instance, when |*ψ*〉 is an eigenstate of either *A* or *B*. Despite of this, the variance-based uncertainty relations possess a clear physical meaning and have variety of applications in the theory of quantum information processing such as entanglement detection^8^,^9^, quantum spin squeezing^10^,^11^,^12^,^13^,^14^, and quantum metrology^15^,^16^,^17^.

Recently Maccone and Pati have presented two stronger uncertainty relations^18^ based on the sum of variances and their inequalities are guaranteed to be nontrivial when |*ψ*〉 is not a common eigenstate of *A* and *B*. Though there are many formulations of the uncertainty relation in terms of the sum of entropic quantities^19^,^20^, Maccone and Pati’s relations capture the notion of incompatibilty except when the state is an eigenstate of the sum of the operators. Their first relation for the sum of the variances is


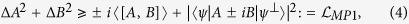


which is valid for any state |*ψ*^⊥^〉 orthogonal to the state of the system |*ψ*〉 while the sign should be chosen so that ±*i*〈[*A*, *B*]〉 is positive. Denote the right-hand (RHS) of Eq. (4) by 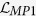. Their second uncertainty relation also provides a nontrivial bound even if |*ψ*〉 is an eigenstate of *A* or *B*:


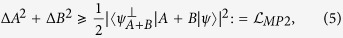


where 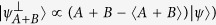 is a state orthogonal to |*ψ*〉. It is easy to see that the RHS 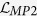 of Eq. (5) is nontrivial unless |*ψ*〉 is an eigenstate of *A* + *B*. Moreover, based on the same techniques, Maccone and Pati also obtained an amended Heisenberg-Robertson inequality:


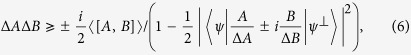


which reduces to Heisenberg-Robertson’s uncertainty relation when minimizing the lower bound over |*ψ*^⊥^〉, and the equality holds at the maximum. The goal of this paper is to give a new method of measuring the uncertainties to remove the restriction on the bounds such as 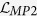.

Actually, both the entropic uncertainty relations and the sum form of variance based uncertainty relations do not suffer from trivial bounds. Generalizing Deutsch’s entropic uncertainty relation^21^, Maassen and Uffink^22^ used certain weighted entropic uncertainties to derive a tighter bound. Adopting a similar idea to the uncertainty relations based on Rényi entropy, we propose a *deformed uncertainty relation* to resolve the restriction of Maccone-Pati’s variance based uncertainty relation. i.e. the new uncertainty relation will provide a nontrivial bound even when the state is an eigenvector of *A* + *B*. Moreover, we show that the original Maccone-Pati’s bound is a singular case in our general uncertainty relation and the usual sum of variances can be extracted from weighted sum of uncertainties. Our work indicates that it seems unreasonable to assume a priori that observables *A* and *B* have equal contribution to the variance-based sum uncertainty relation. Our family of uncertainty relations are proved to possess an optimal bound in various situations according to the state of the system. In particular, all previous important variance-based sum uncertainty relations are special cases of our weighted uncertainty relation.

We remark that there is another approach of *measurement uncertainty*^23^,^24^ to the uncertainty principle which deals with joint measurability and measurement-disturbance. Our methods can also be used to generalize the joint measurability, also known as *preparation uncertainty*^23^, and to obtain a tighter bound.

## Results

We first consider the weighted uncertainty relations based on the sum of variances of two observables, then generalize it into multi-observable cases. All observables considered in the paper will be assumed to be non-degenerate on a finite-dimensional Hilbert space. We will show that our weighted uncertainty relations give optimal lower bounds and all previous important variance-based sum uncertainty relations are special cases of the new weighted uncertainty relation.

**Theorem 1** *For arbitrary observables A, B and any positive number λ, we have the following weighted uncertainty relation:*


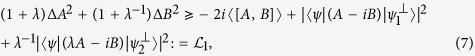


*which is valid for all* 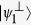 *and* 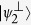 *orthogonal to* |*ψ*〉*. If* −2*i*〈[*A*, *B*]〉 *is negative then one changes its sign in* Eq. (7) *to ensure the RHS is positive*.

The equality condition for Eq. (7) holds if and only if 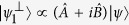 while 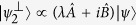. Denote the RHS of Eq. (7) by 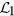. Clearly 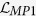 as a special case of 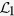, as 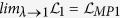. When *λ* varies, one obtains a family of uncertainty relations and the lower bounds 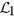 provide infinitely many uncertainty relations with weighted contributions for measurements *A* and *B*. This will be advantageous when the ratio 〈*A*〉/〈*B*〉 is not close to 1.

See Methods for a proof of Theorem 1.

**Theorem 2** *For arbitrary observables A, B and any positive λ, we have the following weighted uncertainty relation:*


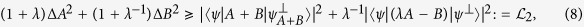


*where the equality holds if and only if* 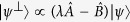.

Denote the RHS of Eq. (8) by 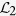. Note that the lower bound 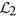 is a nontrivial generalization of 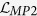, as the latter is a proper bound unless |*ψ*〉 is an eigenstate of *A* + *B*. Even when |*ψ*〉 is an eigenstate of *A* + *B*, the new uncertainty bound 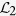 is also nonzero except for *λ* = −1 (Equation (8) still holds for any nonzero real *λ*). This means that in almost all cases the lower bound provided by Eq. (8) is better except for *λ* = −1 and it compensates for the incompatibility of the observables. Obviously the bound 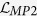 is a special case of 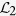 by canceling |〈*ψ*|(*λA* − *B*)|*ψ*^⊥^〉|^2^ when *λ* = 1.

See Methods for a proof of Theorem 2.

Both lower bounds of the weighted uncertainty relations can be combined in a single uncertainty relation for the sum of variances:

**Theorem 3** *For arbitrary observables A, B and any positive number λ, we have the following weighted uncertainty relation*:


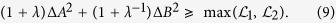


Theorems 1 and 2 provide a strengthened uncertainty relation and remove the limitation of the Maccone-Pati bounds. In fact, in the case when |*ψ*〉 is an eigenstate of *A* or *B*, both Heisenberg-Robertson’s and Schrödinger’s uncertainty relations are trivial, nevertheless our lower bound remains nonzero unless |*ψ*〉 is a common eigenstate of *A* and *B*, but this is essentially equivalent to the classical situation. It is also easy to see that if |*ψ*〉 is an eigenstate of *A* ± *iB*, |〈*ψ*|*A* ± *iB*|*ψ*^⊥^〉|^2^ in 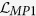 will vanish while the term 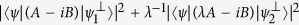 in 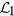 is still nonzero unless *λ* = 1. Moreover, 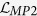 will become null when |*ψ*〉 is an eigenstate of *A* + *B*, but at the same time 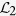 is still nontrivial.

Besides having a nontrivial bound in almost all cases, our weighted uncertainty relations can also lead to a tighter bound for the sum of variances. We give an algorithm to extract the usual uncertainty relation when one of Maccone-Pati’s relations becomes trivial. Choose two *λ*_*i*_: *λ*_1_ > 1 > *λ*_2_ > 0 and enter our uncertainty relations Eq. (7). Denote 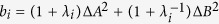, then we have for *k* = 1, 2


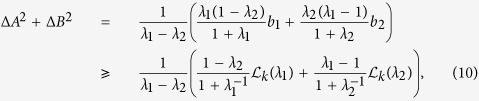


which always provides a nontrivial lower bound for the sum of variances even when the state is an eigenvector of *A* + *B*. This clearly shows that the weighted uncertainty relations can help recover the uncertainties and remove the restriction placed in Maccone-Pati’s uncertainty relation. Furthermore, taking the limit of *λ*_*i*_ → 1 one has that for *k* = 1, 2


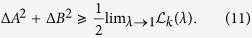


**Figure 1:**
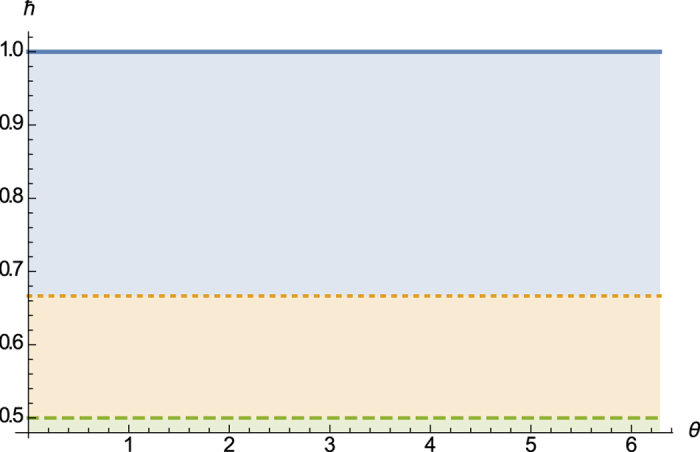
Comparison of our bound 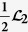 with Maccone-Pati’s bound 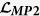 for operators *J*_*x*_ and *J*_*y*_ in a spin one system. The top solid line is variance sum uncertainty (Δ*J*_*x*_)^2^ + (Δ*J*_*y*_)^2^, the middle dotted line is 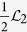, and the bottom dashed one is 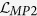.

For simplicity we refer to the RHS of Eq. (10) or the derived bound in Eq. (11) as *our lower bound of the sum of variances*, which usually is a multiple of our bound from the weighted sum (see Fig. 1). In Fig. 1 one will see that our bound 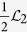 derived in Eq. (11) is alwasy tighter than the Maccone-Pati bound 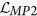. In Eq. (14) we will use another method to show that our bound is tighter than Maccone-Pati’s bound.

As an example to show our lower bound is tighter, we consider the spin one system with the pure state 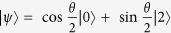, 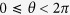. Take the angular momentum operators^25^,^26^ with *ħ* = 1:


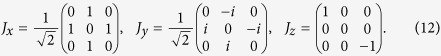


Direct calculation gives


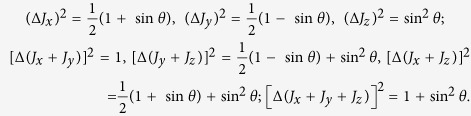


To compare Macconne-Pati’s uncertainty bound 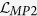 in Eq. (5) with our bound 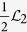 in Eq. (8) (see also Equation (11)), setting *λ* = 1 we get


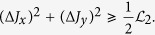


Also we have (Δ*J*_*x*_)^2^ + (Δ*J*_*y*_)^2^ = 1 and 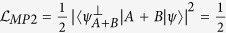. Suppose |*ψ*^⊥^〉 = *a*|0〉 + *b*|1〉 + *c*|2〉 with |*a*|^2^ + |*b*|^2^ + |*c*|^2^ = 1. Using 〈*ψ*|*ψ*^⊥^〉 = 0 we get


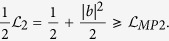


If we choose 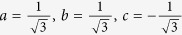, then 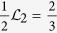. Subsequently


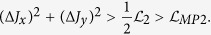


On the other hand, if we set *a* = 0, *b* = 1, *c* = 0 then 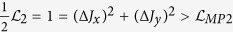. Clearly our bound 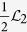 is tighter than 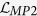. The comparison is shown in Fig. 1.

We can also consider (Δ*J*_*y*_)^2^ + (Δ*J*_*z*_)^2^, and direct computation shows 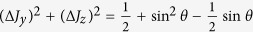, 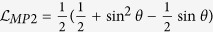. Choose |*ψ*^⊥^〉 = |1〉 then 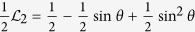. Therefore


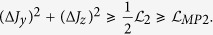


**Figure 2:**
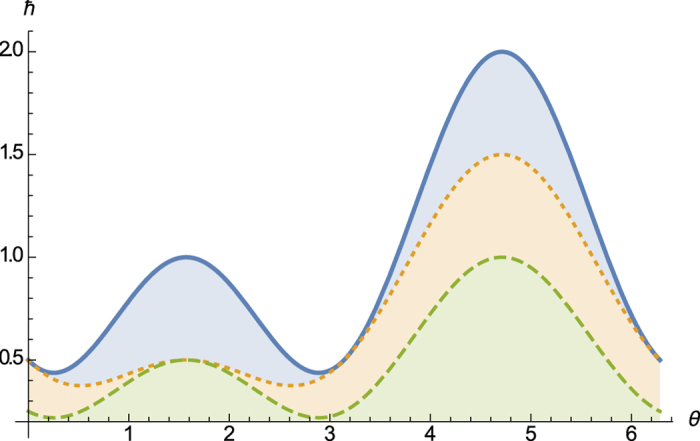
Comparison of our bound 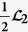 with Maccone-Pati’s bound 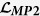 for operators *J*_*y*_ and *J*_*z*_ in a spin one system. The top solid curve is variance sum uncertainty (Δ*J*_*y*_)^2^ + (Δ*J*_*z*_)^2^, the middle dotted curve is 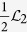 and the bottom dashed one is 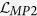.

Apparently our bound 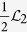 is better than 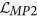. Figure 2 illustrates the comparison.

The bound 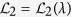 is a function of *λ*. To analyze when 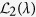 best approximates (1 + *λ*)Δ*A*^2^ + (1 + *λ*^−1^)Δ*B*^2^, we define *the error function* 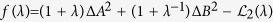. At an extremal point *λ*_0_, the bound 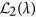 is closest to the weighted sum and one of the following two conditions must hold. Either *f* ′(*λ*_0_) does not exist or 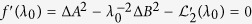. If 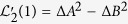, then *λ* = 1 is the extremal point and we call it an *equilibrium point of the uncertainty relation*. In this case both observables *A* and *B* give the same contribution to the uncertainty relation. Usually *λ* = 1 is not an extremal point, so in general observables *A* and *B* contribute unequally to the uncertainty relation.

**Figure 3:**
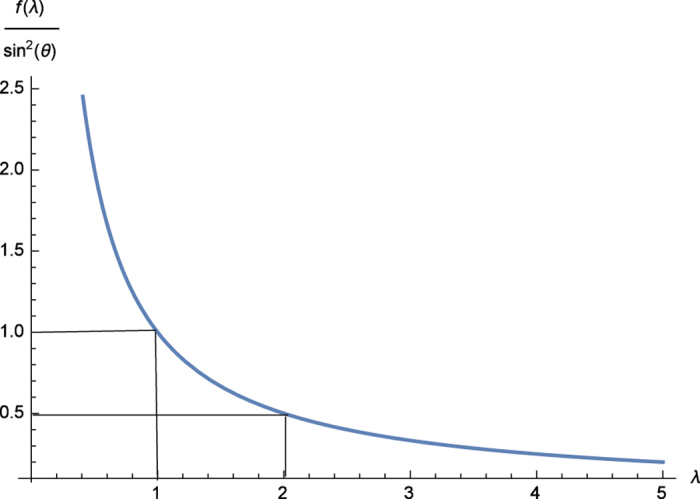
Error function Eq. (13) of Uncertainty Relation. The figure shows that the difference between uncertainty relation and its bound for fixed form 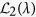 becomes less when *λ* increases, which means that better estimation may be obtained through larger *λ*.

To see an example of this phenomenon, let’s consider again the quantum state 
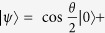

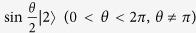
 and the angular momentum operators. Choose |*ψ*^⊥^〉 = |1〉, then


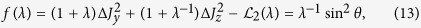


while 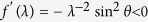, hence *f*(1) > *f*(*λ*), ∀*λ* > 1 (for fixed *θ*). So for this |*ψ*〉, *J*_*y*_ and *J*_*z*_ never contribute equally to the uncertainty relation, which explains the need for a weighted uncertainty relation. Figure 3 shows the error function *f*(*λ*) and 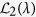. In general *f* is a function of both *λ* and *θ*, finding its extremal points involves a PDE equation. For higher dimension quantum states or multi-operator cases, the situation is more complicated.

In general, all variance-based sum uncertainty relations can mix in weights to provide an optimal lower bound. To compare the variance-based sum uncertainty relation with weighted uncertainty relation, take the lower bound 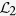 for a more detailed analysis: set *λ* = 1 then 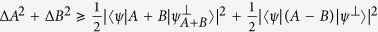, it is not only a typical variance-based sum uncertainty relation, but also provides a better lower bound than Maccone-Pati’s lower bound 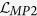. Moreover, this lower bound can be further improved by a mixture of weights.

**Corollary 1** *For arbitrary observables A, B and any positive number λ, we have the following weighted uncertainty relation:*


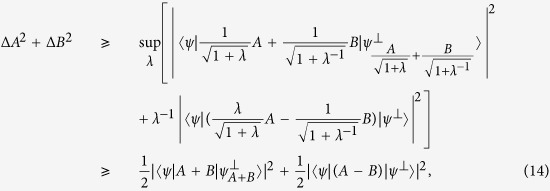


*where* 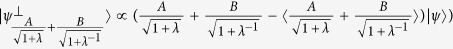 *is a state orthogonal to* |*ψ*〉.

Through Eq. (14), it is easy to see 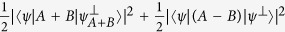 is the special case of *λ* = 1 and, *a fortiori*, the lower bound with weights is tighter than the standard one.

See Methods for a proof of Corollary 1.

One can study the general weighted sum of variances *x*Δ*A*^2^ + *y*Δ*B*^2^ based on the special weighted sum (1 + *λ*)Δ*A*^2^ + (1 + *λ*^−1^)Δ*B*^2^. Theorem 4 details the relationship between the general and special weighted sum uncertainty relations.

**Theorem 4** *For arbitrary observables A, B and x, y such that xy*(*x* + *y*) > 0*, the following weighted uncertainty relation holds*.


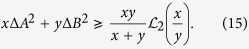


See Methods for a proof of Theorem 4.

According to Deutsch^21^, uncertainty in the result of a measurement of observables *A* and *B* should be quantified as an inequality with certain lower bound. One can seek such a bound in a general form 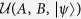 which may not simply be a sum or product by weighted uncertainty relations. For instance, we take 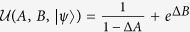, its bound can be extracted from Theorem 4.

**Remark 1** *For* |Δ*A*| < 1 *and arbitrary observable B*, 
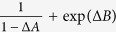
 *has a nonnegative lower bound:*


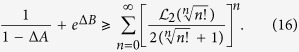


See Methods for a proof of Remark 1.

We now generalize the weighted uncertainty relations to multi-operator cases. To emphasize our point, we recall the trivial generalization from Maccone-Pati’s lower bound.

**Lemma 1** *For arbitrary observables A*_*i*_ *(i* = 1, …, *n), we have the following variance-based sum uncertainty relation:*


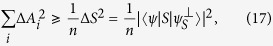


*where* 
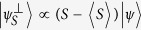
 *is a unit state perpendicular to* |*ψ*〉 *while* 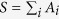
*. The RHS of* Eq. (17) is nonzero unless |*ψ*〉 is an eigenstate of 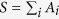.

See Methods for a proof of Lemma 1.

Notice that |*ψ*〉 can be an eigenstate of 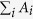 without being that of any *A*_*i*_, in which case the lower bound is still trivial. However, the bound is not optimal and sometimes becomes trivial when the observables are incompatible in the general situation. We now introduce *generalized weighted uncertainty relations* to deal with these drawbacks.

**Theorem 5** *For arbitrary n observables A*_*i*_ *and positive numbers λ*_*i*_*, we have following sum uncertainty relation:*


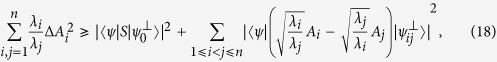


*where* 
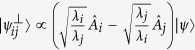
 *and*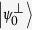 *is any unit state* ⊥|*ψ*〉.

See Methods for a proof of Theorem 5.

The RHS 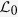 of (18) depends on the choice of *λ*_*i*_. By the same trick and fixing the (*i*, *j*)-term of Eq. (18), we arrive at

**Theorem 6** *For arbitrary n observables A*_*i*_ *and positive numbers λ*_*i*_*, we have following sum uncertainty relation:*


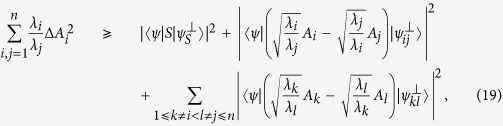


*where* 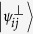 *is orthogonal to* |*ψ*〉, 
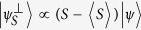
*, and*
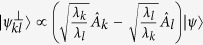
.

Clearly, 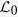 and all the RHS 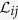 of Eq. (19) comes form Theorem 5. and Theorem 6. respectively can be combined into a single uncertainty relation for variances:

**Theorem 7** *For arbitrary n observables A*_*i*_ *and any positive numbers λ*_*i*_*, we have the following sum uncertainty relation:*


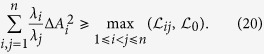


When setting *λ*_*i*_ = *λ*_*j*_, the RHS of Eq. (20) is still stronger than Eq. (17), since it keeps all the terms 
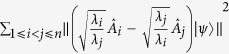
 appearing in Eq. (18). We remark that a default choice of |*ψ*^⊥^〉 in Eq. (19) is by Vaidman’s formula^27^,^28^: 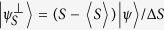. We can select suitable *λ*_*i*_ such that 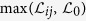 is nontrivial. They are zero if and only if |*ψ*〉 is a common eigenstate of all observables, which happens only when the system is equivalent to the classical situation. In this sense our weighted uncertainty relation can handle all possible quantum situations.

If two or more terms in the RHS of these equality are replaced by the Cauchy-Schwarz’s inequality simultaneously, the corresponding lower bound can not be bigger than the one by replacing just one term. In other words, 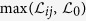 is better than the lower bounds by changing more than one term. The LHS of Eq. (20) has only positive coefficients since *λ*_*i*_ are positive.

## Discussions

There are several physical motivations and mathematical considerations behind our method. First, to remove the restriction of one of Macconne-Pati’s uncertainty relations (i.e. when *ψ* is an eigenstate of *A* + *B*) and recover the lower bound for Δ^2^(*A*) + Δ^2^(*B*), we consider a perturbation of *A* and *B*, or rather, 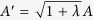, 
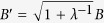
 (*λ* > 0). Then


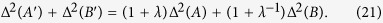


This means that the lower bound of the sum of variances can be obtained by scaled observables. Actually with the given measurement data of the variances, it is easy to compute the lower bound using our new formula. This is in line with the general strategy of perturbation method, just as many singular properties can be better studied through deformation.

Secondly, the idea of the weighted sum or average is similar to well-known techniques used in both statistical mechanics and mathematical physics. Through the weighted averages one may know better about the whole picture in an unbiased way.

Thirdly, the weighted sum is actually a *q*-deformation of the original sum of variances. In fact, the sum 2Δ^2^(*A*) + 2Δ^2^(*B*) is deformed to


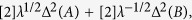


where [2] = *λ*^1/2^ + *λ*^−1/2^ is the quantum integer of 2 used widely in quantum groups, Yang-Baxter equations, and quantum integrable systems or statistical mechanics. The opposite phase factors *λ*^±1/2^ in front of the variances reflect a balance of the weighted distribution.

Last but not the least, the usual sum of variances can be solved from our weighted sums (see Eqs (10, 11)), and the derived bound is proved to be tighter than the original bound of Maccone-Pati’s bound.

## Conclusions

The Heisenberg-Robertson and Schrödinger uncertainty relations have been skillfully generalized by Maccone and Pati in order to capture the concept of incompatibility of the observables *A* and *B* on the quantum system |*ψ*〉. Although other generalizations of Maccone-Pati’s relations have been considered^29^ by refining the RHS, our generalization provides a non-trivial lower bound in all quantum situations. One of Maccone-Pati’s relations becomes trivial when |*ψ*〉 is an eigenstate of *A* + *B*. To remove the restriction of their relation, we have proposed a weighted uncertainty relation to obtain a better lower bound for the sum of the variances. The parametric uncertainty relations form a family of Bohr-type inequalities and take into account of individual contribution from the observables so that they are nontrivial in almost all cases except when |*ψ*〉 is a common eigenstate of all observables. In particular, Maccone-Pati’s uncertainty relations are special cases of our deformed weighted uncertainty relations. Furthermore, we have shown that the sum of variances can be extracted from our weighted sums and our derived bound is always tighter than Maccone-Pati bound 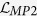 (see discussion before Equation (11)). We have also derived weighted uncertainty relations for multi-observables and the lower bound has been proved to be optimal in all quantum cases.

## Methods

**Proof of Theorem 1** We start by recalling the parallelogram law in Hilbert space. Let *A* and *B* be two observables and |*ψ*〉 a fixed quantum state. One has that


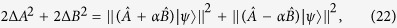


for any |*α*| = 1. Since 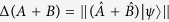, 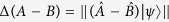, we can obtain Eq. (4) when *α* = ±*i* and Eq. (5) when *α* = 1. Note that 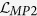 may be zero even if *A* and *B* are incompatible. For example this happens if |*ψ*〉 is an eigenstate of *A* + *B*. Our idea is to consider a perturbation of *A* + *B*, or *A* and *B* to fix this. We consider the generalized parallelogram law in Hilbert space in the following form:


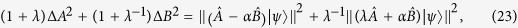


where *λ* is a nonzero real number and 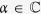 with modulus one. In fact, the identity can be easily verified by expanding Δ(*A* − *αB*)^2^ and *λ*^−1^Δ(*λA* + *αB*)^2^ using 
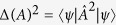
.

We now derive the *weighted uncertainty relation* in the form (1 + *λ*)Δ*A*^2^ + (1 + *λ*^−1^)Δ*B*^2^. Since 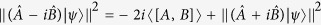, combine with Cauchy-Schwarz inequality completes the proof.

**Proof of Theorem 2** If we set *α* = −1 in Eq. (23), then we get the result directly.

**Proof of Corollary 1** For *λ* > 0, set 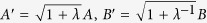 (see Equation (21)), so


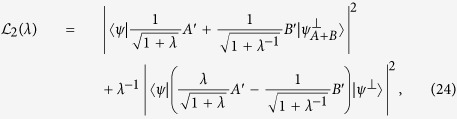


where the RHS 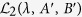 satisfies that 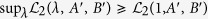 which implies that the weighted uncertainty relation is better than the ordinary sum: 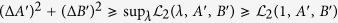. Followed by parameter transformation, we get Eq. (14).

**Proof of Theorem 4** For arbitrary weighted uncertainty relation *x*Δ*A*^2^ + *y*Δ*B*^2^, denote 
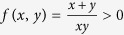
, then


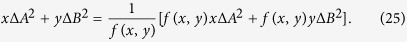


Set 
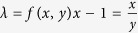
, then *λ*^−1^ = *f*(*x*, *y*)*y* − 1. Thus


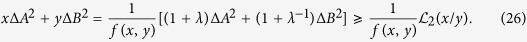


**Proof of Remark 1** Since


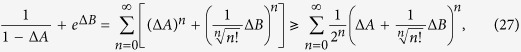


with *x* = 1, 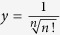, 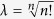 and 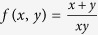, we get


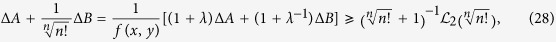


thus


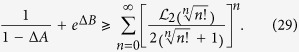


The right-hand is a positive lower bound of uncertainty relation 
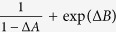
.

**Proof of Lemma 1** We recall Maccone-Pati’s lower bound 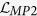 using a different method.

Note that 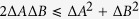 and 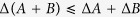, therefore 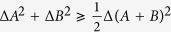. The physical meaning is that the total ignorance of an ensemble of quantum states is less than or equal to the sum of individual ignorance. This means that the sum of uncertainties obeys the convexity property^30^:


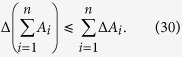


Let 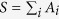. It follows from Eq. (30) that


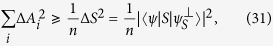


where 
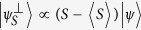
 is a unit state perpendicular to |*ψ*〉.

**Proof of Theorem 5** Using the generalized parallelogram law and Bohr’s inequality^31^,^32^,^33^,^34^,^35^,^36^, we obtain the following relation:


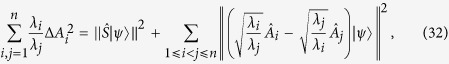


where 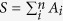, 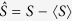 and *λ*_1_, …,*λ*_*n*_ are positive real numbers. Combining with Cauchy-Schwarz inequality, we derive Eq. (18).

## Additional Information

**How to cite this article**: Xiao, Y. *et al*. Weighted Uncertainty Relations. *Sci. Rep*. **6**, 23201; doi: 10.1038/srep23201 (2016).
